# Attenuation of cardiac hypertrophy by G-CSF is associated with enhanced migration of bone marrow-derived cells

**DOI:** 10.1111/jcmm.12494

**Published:** 2015-03-08

**Authors:** Bruno C Huber, Nick L Beetz, Alexandra Laskowski, Tilman Ziegler, Ulrich Grabmaier, Christian Kupatt, Nadja Herbach, Ruediger Wanke, Wolfgang-Michael Franz, Steffen Massberg, Stefan Brunner

**Affiliations:** a Medical Department I, Campus Grosshadern and Campus Innenstadt, Ludwig-Maximilians-UniversityMunich, Germany; b Institute of Veterinary Pathology, Ludwig-Maximilians-UniversityMunich, Germany; c Department of Internal Medicine III, Cardiology and Angiology, Medical University InnsbruckInnsbruck, Austria

**Keywords:** G-CSF, hypertrophy, BMCs, migration, fibrosis

## Abstract

Granulocyte-colony stimulating factor (G-CSF) has been shown to promote mobilization of bone marrow-derived stem cells (BMCs) into the bloodstream associated with improved survival and cardiac function after myocardial infarction. Therefore, the aim of the present study was to investigate whether G-CSF is able to attenuate cardiac remodelling in a mouse model of pressure-induced LV hypertrophy focusing on mobilization and migration of BMCs. LV hypertrophy was induced by transverse aortic constriction (TAC) in C57BL/6J mice. Four weeks after TAC procedure. Mice were treated with G-CSF (100 μg/kg/day; Amgen Biologicals) for 2 weeks. The number of migrated BMCs in the heart was analysed by flow cytometry. mRNA expression and protein level of different growth factors in the myocardium were investigated by RT-PCR and ELISA. Functional analyses assessed by echocardiography and immunohistochemical analysis were performed 8 weeks after TAC procedure. G-CSF-treated animals revealed enhanced homing of VLA-4^+^ and c-kit^+^ BMCs associated with increased mRNA expression and protein level of the corresponding homing factors Vascular cell adhesion protein 1 and Stem cell factor in the hypertrophic myocardium. Functionally, G-CSF significantly preserved LV function after TAC procedure, which was associated with a significantly reduced area of fibrosis compared to control animals. Furthermore, G-CSF-treated animals revealed a significant improvement of survival after TAC procedure. In summary, G-CSF treatment preserves cardiac function and is able to diminish cardiac fibrosis after induction of LV hypertrophy associated with increased homing of VLA-4^+^ and c-kit^+^ BMCs and enhanced expression of their respective homing factors VCAM-1 and SCF.

## Introduction

LV hypertrophy accompanied by interstitial myocardial fibrosis is one of the main reasons for heart failure in patients with arterial hypertension and aortic stenosis (AS) 1. Moreover, it has been shown that cardiac fibrosis is an important morphological substrate of postoperative clinical outcome in patients with severe AS and is not completely reversible after aortic valve replacement 2. No medical therapies are particularly effective for retarding and reversing this maladaptive remodelling in patients with LV hypertrophy 3. Therefore, there is a great interest in new strategies to qualitatively and quantitatively alter collagen production and turnover, as well as to modify different extracellular matrix (ECM) components, which are crucial in mediating myocardial hypertrophy and fibrosis 4.

In the last years, regeneration of ischaemic myocardial tissue has been attempted using bone marrow-derived stem cells (BMCs) and pluripotent stem cells 5,6. As an alternative to the direct intramyocardial injection 7,8, mobilization of BMCs using growth factors, such as the hematopoietic cytokine granulocyte-colony stimulating factor (G-CSF), have been successfully utilized 9–11. Migration of circulating mobilized BMCs from the bloodstream into the damaged tissue is suggested to play a key role for tissue regeneration 12. The interaction of stromal cell-derived factor-1 (SDF-1/CXCL12) with its ligand chemokine receptor type 4 (CXCR4) on mobilized hematopoietic progenitor cells has been shown to play a major role in migration of BMCs into ischaemic myocardium 11,13. G-CSF administration after acute myocardial infarction (MI) resulted in diminished stem cell homing caused by the N-terminal cleavage of SDF1 14 and this could be one explanation for the poor clinical results of patients treated with G-CSF post MI 15. However, in non-ischaemic cardiomyopathy the interaction of SDF-1 with CXCR4 seems not to be involved in a cardiac self-repair mechanism, because cardiac SDF-1 expression was not up-regulated in dilated cardiomyopathy (DCM) hearts of humans 16.

There is first evidence that G-CSF might be a promising therapeutic agent to treat congestive heart failure because of cardiac hypertrophy and fibrosis. Szardien *et al*. were able to demonstrate that G-CSF leads to the regression of myocardial fibrosis and improves cardiac function after pressure unloading in mice 17. However, the exact mechanisms of fibrosis regression after G-CSF treatment are still unclear.

Therefore, the aim of our study was to investigate whether G-CSF is able to attenuate cardiac hypertrophy and fibrosis in a mouse model of pressure-induced LV hypertrophy induced by transverse aortic constriction (TAC). Moreover, we wanted to exploit whether and how G-CSF is facilitating enhanced migration of BMCs after TAC procedure. To avoid impairing the cellular setting of this hypertrophy model and to ensure all findings are caused by the treatment only, we did not perform pressure unloading.

## Material and methods

### Animal model

As a model for pressure-induced LV hypertrophy, C57Bl/6 wild-type mice (Charles River Laboratories, Sulzbach, Germany) were subjected to TAC. All mice used in this study were 8–10 weeks old healthy males. Mice were anesthetized by an intra-peritoneal (i.p.) injection of a mixture of Medetomidine (Domitor®, Pfizer, Berlin, Germany), Midazolam (Midazolam ratiopharm®, Ratiopharm, Ulm, Germany) and Fentanyl (Fentanyl-Janssen®, Janssen-Cilag, Neuss, Germany). Mice were placed in supine position on a heating pad and temperature was maintained at 37°C. For the surgical procedure mice were intubated, and artificially ventilated by a mouse ventilator (MiniVent, HUGO SACHS, March, Germany) with 200 strokes/min. and 200 μl/stroke. A short skin cut longitudinal to the sternum was followed by a 3–4 mm long sectioning of the cranial sternum. The thymus lobes were separated and secured by two retractors, giving a free view of the aortic arch. Between the right brachiocephalic artery and the left common carotid artery, the transverse aorta was carefully mobilized by forceps, allowing to navigate a silk thread (6-0 polypropylene suture) behind the aorta. For constriction, a 27-gauge needle was placed between transverse aorta and silk thread before instrument tying. The removal of the needle ensured a standardized diameter of 0.4 mm of the transverse aorta. The sternum was adapted with a 5-0 polypropylene suture. The skin was closed with a 5-0 silk suture and mice were allowed to recover from anaesthesia on the heating pad. Animal care and all experimental procedures were performed in strict accordance to the German and National Institutes of Health animal legislation guidelines and were approved by the local animal care committees (Regierung von Oberbayern). The investigation conforms to the Guide for the Care and Use of Laboratory Animals published by the US National Institutes of Health (NIH Publication No. 85-23, revised 1996).

### Administration of G-CSF

4 weeks after TAC procedure, mice were randomly divided into the following groups, each treated for 2 weeks, receiving five injections per week: (*i*) subcutaneous administration of saline (0.9% NaCl) and (*ii*) administration of G-CSF (100 μg/kg/day; Amgen Biologicals, Thousand Oaks, CA, USA).

### Flow cytometry of non-myocyte cardiac cells

For analysis of BMCs within the hearts, a ‘myocyte-depleted’ cardiac cell population was prepared, incubating minced myocardium in 0.1% collagenase IV (GIBCO BrL, Carlsbad, CA, USA) 30 min. at 37°C, lethal to most adult mouse CMs. Cells were then filtered through a 70 mm mesh. To exclude spurious effects of enzymatic digestion, BM cells with or without collagenase treatment were stained revealing no significantly changed staining of labelled cell antigens. Mononuclear cells were separated by density-gradient centrifugation using 1.077 g/ml Histopaque solution (Sigma Chemicals, St. Louis, MO, USA), purified, and resuspended in PBS containing 1% BSA. Cells were incubated for 40 min. in the dark at 4°C with the following fluoresceinisothiocyanate (FITC), phycoerythrin (PE) and peridininchlorophyll-protein (PerCP) conjugated monoclonal antibodies: CD45-PerCP, CD34-FITC, VLA-4-PE, c-kit-PE and CXCR4-PE (all from BD Pharmingen). Matching isotype antibodies (BD Pharmingen, San Jose, CA, USA) served as controls. Cells were analysed by three-colour flow cytometry using a Coulter® Epics®XL-MCLTM flow cytometer (Beckman Coulter, Cincinnati, OH, USA). Each analysis included 50.000 events.

### Quantitative RT-PCR and ELISA

Hearts were explanted and the LVs were separated. Isolation of total RNA from mouse heart tissue was performed with TRI REAGENT (Molecular Research Center, Inc., Cincinnati, OH, USA) according to the manufacturer's protocol. Reverse transcription was performed with the ImProm-IITM Reverse Transcription System (Promega, Fitchburg, WI, USA) according to the manufacturer's protocol. cDNA samples were analysed by quantitative RT-PCR using adequate murine primers purchased from MWG-BIOTECH AG, Ebersberg, Germany. Quantitative RT-PCR was performed with SYBR Green Reaction Mix (Eurogentec, Cologne, Germany) on an ABI PRISM 7900HT Detection System (Applied Biosystems, Carlsbad, Germany). Each sample was run in duplicate. The expression of each gene within the different tissue samples was quantified relative to H4 mRNA expression levels according to the Sequence Detector User Bulletin (Applied Biosystems).

VCAM-1 and SCF protein levels were quantified using commercially available kits (R&D Systems, Minneapolis, MN) according to the manufacturer's instructions.

### Histopathology

8 weeks after the TAC procedure, hypertrophic hearts were extracted after perfusion fixation *via* blood flow. Before punctuating the right ventricle, hepatic veins were cut. First, the hearts were cleaned from blood with PBS buffer for 1 min. Then perfusion fixation started with formalin (4%) for 5 min. After carefully dissecting the LV, it was embedded in formalin (4%) for 24 hrs and afterwards in paraffin. 5 μm thick sections were stained with hematoxilin/eosin and Masson's trichrome. Fibrosis was measured using a blinded counting system. For detection of apoptotic nuclei, a terminal desoxynucleotidyl transferase dUTP nick end labelling (TUNEL) was used, allowing to identify DNA fragmentation.

### Echocardiographic imaging

Transthoracic echocardiography was performed with a Vevo2100 imaging system (Visual Sonics, Toronto, ON, Canada) using a 40 MHz transducer. Therefore, mice were placed on a heating pad, maintaining a body temperature of 37°C and providing ECG-triggered analysis of heart function. After chemical depilation of the chest, mice were prepared for echocardiography with ultrasound gel. Mice were sedated with 2% isoflurane in 100% O_2_ at all times. Ejection fraction (EF) was evaluated by planimetric measurement in the parasternal long-axis (Simpson method). For evaluation of EF, three consecutive cycles were measured and averaged. Diastolic thickness of the LV posterior wall (LVPW) was measured in the parasternal short-axis. The measurements were performed twice by two blinded investigators.

### Statistical analysis

Data are shown as mean ± SEM. Multiple group comparison was performed by one-way anova followed by the Bonferroni procedure for comparison of means. Comparisons between two groups were performed with the unpaired *t*-test. Analysis of survival was performed by the Kaplan–Meier method. Between-group difference in survival was tested with the log-rank test. Values of *P* < 0.05 were considered statistically significant.

## Results

### BMCs in hearts of mice with induced cardiac hypertrophy

**Figure 1:**
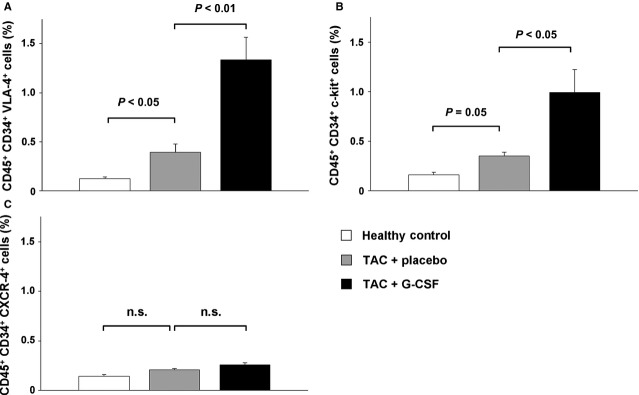
Bone marrow-derived progenitor cells in the heart of healthy control mice (white bar), placebo treated mice with induced cardiac hypertrophy (TAC + placebo, grey bar) and G-CSF treated mice with induced cardiac hypertrophy (TAC + G-CSF, black bar). (A) CD45^+^ CD34^+^ VLA-4^+^ cells. (B) CD45^+^ CD34^+^ c-kit^+^ cells. (C) CD45^+^ CD34^+^ CXCR4^+^ cells. All cell populations are presented in % of mononuclear cells in the heart.

To analyse the influence of cardiac hypertrophy on migration of BMCs (CD45^+^/CD34^+^ cells), we performed flow cytometry of a myocyte-depleted fraction of cardiac cells. In mice with cardiac hypertrophy the number of migrated CD45^+^ CD34^+^ VLA-4^+^ and CD45^+^ CD34^+^ c-kit^+^ cells was significantly increased compared to healthy control mice (Fig.1A and B), whereas the number of CD45^+^ CD34^+^ CXCR4^+^ cells showed no difference (Fig.1C). G-CSF treatment in mice with cardiac hypertrophy further markedly increased the number of CD45^+^ CD34^+^ VLA-4^+^ cells (3.4-fold; *P* < 0.01) and CD45^+^ CD34^+^ c-kit^+^ cells (2.8-fold; *P* < 0.05; Fig.1A and B). The number of CD45^+^ CD34^+^ CXCR4^+^ cells was not affected by the treatment with G-CSF (Fig.1C).

### Expression of myocardial homing factors in mice with induced cardiac hypertrophy

**Figure 2:**
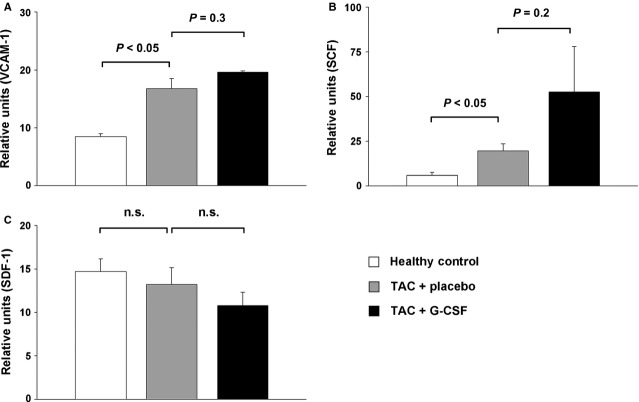
Expression of homing factors in the heart of healthy control mice (white bar), placebo treated mice with induced cardiac hypertrophy (TAC + placebo, grey bar), and G-CSF treated mice with induced cardiac hypertrophy (TAC + G-CSF, black bar). (A) mRNA expression levels of VCAM-1. (B) mRNA expression levels of SCF. (C) mRNA expression levels of SDF-1.

To investigate potential migration pathways involved in cardiac hypertrophy, we analysed murine hearts for the expression of myocardial homing factors (VCAM-1, SCF, SDF-1) interacting with the measured subpopulations of BMCs using quantitative RT-PCR. The expression levels of VCAM-1 (interacting with VLA-4) and SCF (interacting with c-kit) were significantly up-regulated in mice with induced cardiac hypertrophy compared to healthy controls (Fig.2A and B). The expression level of SDF-1 (interacting with CXCR4) showed no difference between mice with cardiac hypertrophy and healthy controls (Fig.2C). G-CSF treatment in mice with cardiac hypertrophy showed a further increase of VCAM-1 and SCF, but without significance (Fig.2A and B). G-CSF treatment did not affect the expression levels of SDF-1 (Fig.2C).

### Protein levels of VCAM-1 and SCF in mice with induced cardiac hypertrophy

**Figure 3:**
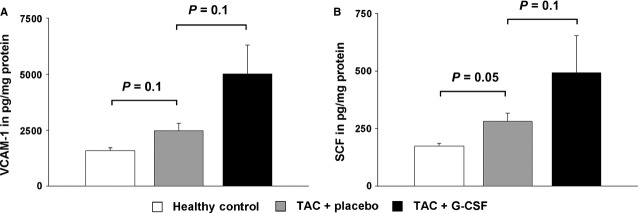
Protein levels in the heart of healthy control mice (white bar), placebo treated mice with induced cardiac hypertrophy (TAC + placebo, grey bar), and G-CSF treated mice with induced cardiac hypertrophy (TAC + G-CSF, black bar) analysed by ELISA. (A) Protein levels of VCAM-1. (B) Protein levels of SCF.

To confirm these findings, we analysed VCAM-1 and SCF on protein levels using ELISA. VCAM-1 and SCF concentrations were increased in mice with cardiac hypertrophy compared to healthy control mice. G-CSF treatment showed a further increase in protein levels of VCAM-1 and SCF in murine hearts (Fig.3).

### Impact of G-CSF therapy on myocardial fibrosis and apoptosis in mice with cardiac hypertrophy

**Figure 4:**
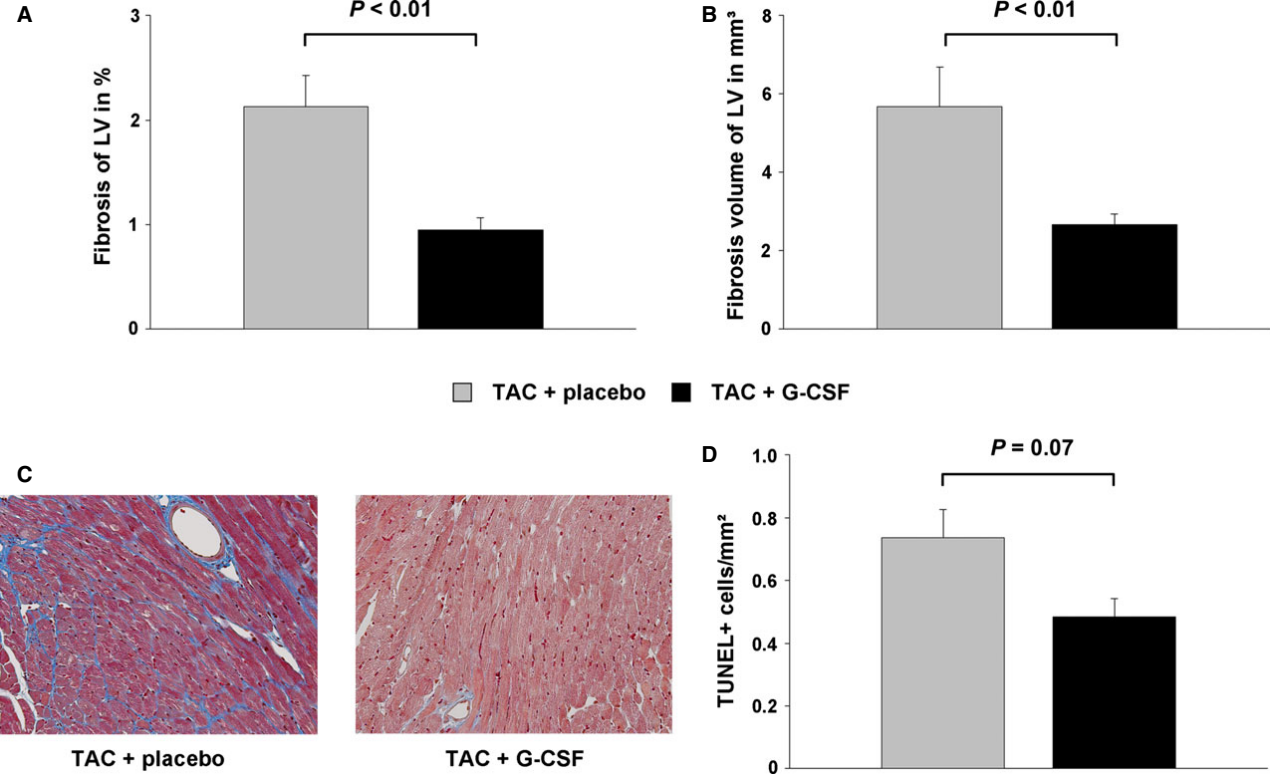
Intramyocardial fibrosis and apoptosis in the LV analysed by histology in placebo treated mice with induced cardiac hypertrophy (TAC + placebo, grey bar), and G-CSF treated mice with induced cardiac hypertrophy (TAC + G-CSF, black bar). (A) Fibrosis of the LV in %. (B) Fibrosis volume of the LV in mm^3^. C Representative histological images of placebo treated mice (left) and G-CSF treated mice (right). (D) Number of TUNEL-positive cells/mm^−2^.

To visualize the impact of G-CSF on myocardial fibrosis and apoptosis in cardiac hypertrophy, the murine hearts were analysed by histology. The percentage of intramyocardial fibrosis of the LV was significantly reduced in G-CSF treated mice (0.95 ± 0.11% *versus* 2.13 ± 0.30%; *P* < 0.01; Fig.4A). The fibrosis volume of the LV, expressed in mm^3^, was also significantly reduced in G-CSF treated mice (2.67 ± 0.27 mm^3^ *versus* 5.67 ± 1.02 mm^3^; *P* < 0.01; Fig.4B and C). The number of TUNEL-positive nuclei was reduced in G-CSF treated mice (0.49 ± 0.06 TUNEL^+^ cells/mm^2^, *versus* 0.74 ± 0.09 TUNEL^+^ cells/mm^2^), although the difference was not significant (*P* < 0.07; Fig.4D).

### Echocardiographic parameters in mice with cardiac hypertrophy

**Figure 5:**
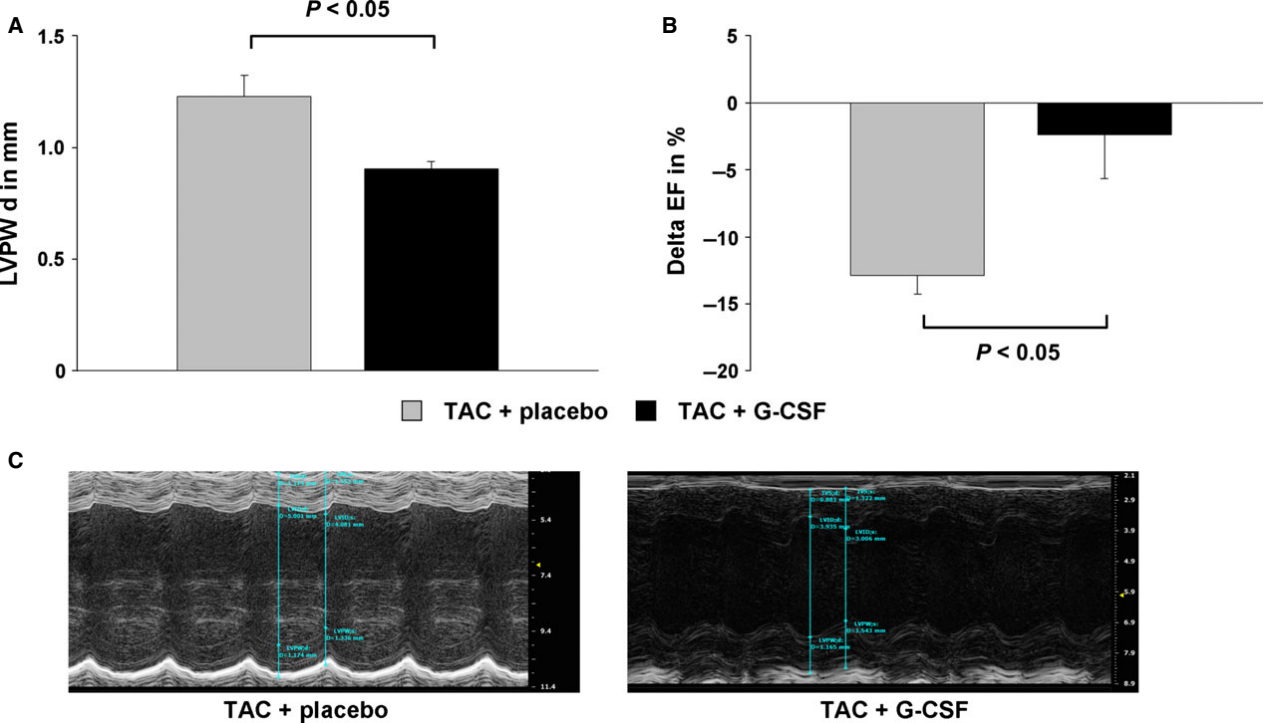
Echocardiographic results in placebo treated mice with induced cardiac hypertrophy (TAC + placebo, grey bar), and G-CSF treated mice with induced cardiac hypertrophy (TAC + G-CSF, black bar). (A) Enddiastolic diameter of the LV posterior wall (LVPW d) after treatment with placebo or G-CSF. (B) Difference of ejection fraction (EF) before and after treatment with placebo or G-CSF expressed as Delta EF. (C) Representative echocardiogram (M-mode) of mice after TAC procedure and treatment with placebo or G-CSF.

Cardiac parameters were assessed by echocardiography before and after G-CSF/placebo-treatment. The LVPW thickness was significantly reduced in G-CSF treated mice compared to the control group (−26%; *P* < 0.05; Fig.5A). EF in the G-CSF treated group decreased by 2.37% ± 3.31% (Delta-EF concerning the difference of EF before treatment and EF 4 weeks later). In contrast, EF in the control group decreased by 12.85% ± 1.40% (Delta-EF; Fig.5B and C).

### Cumulative survival 4 weeks after initiation of treatment

**Figure 6:**
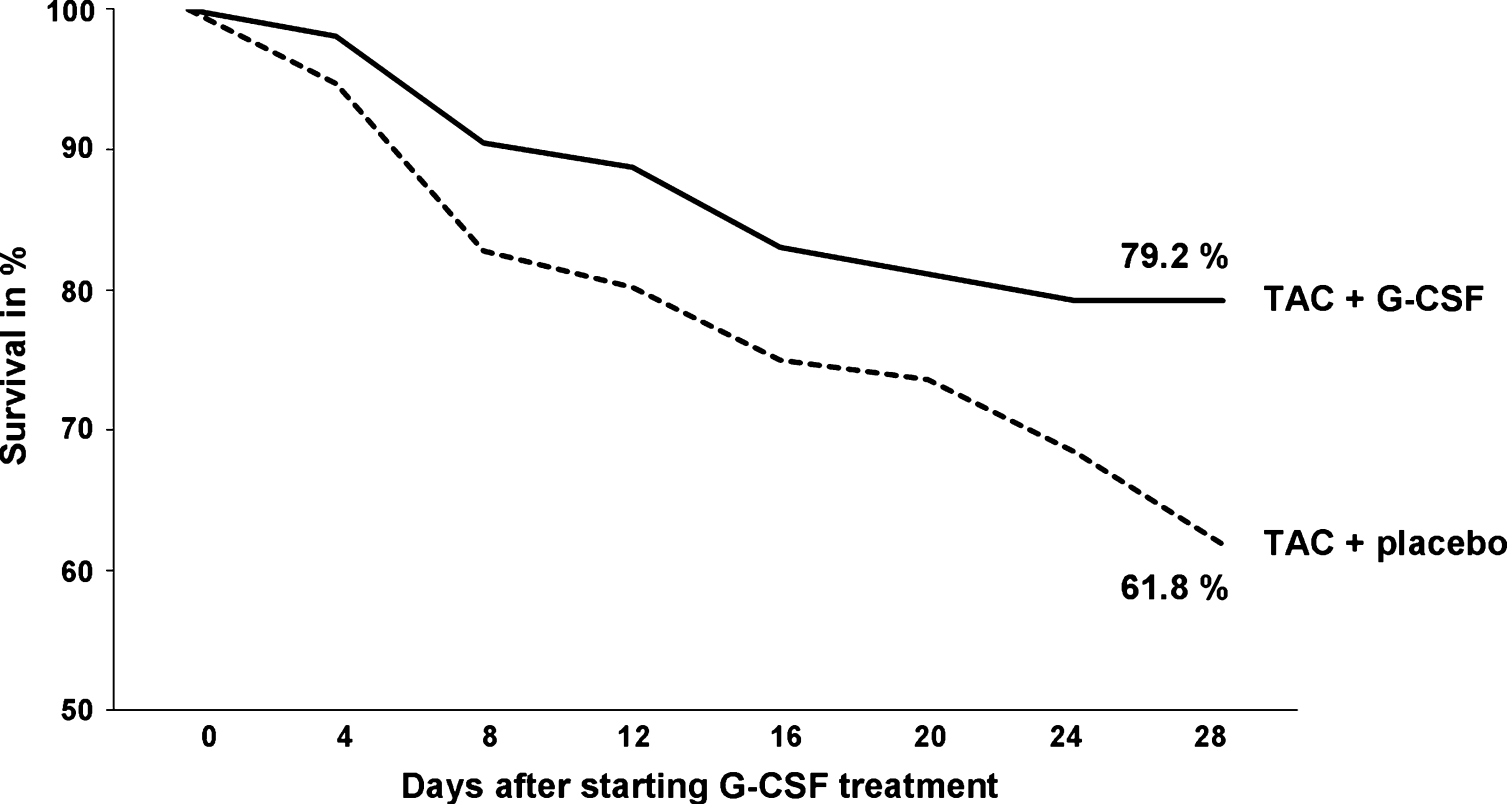
Cumulative survival of placebo treated mice with induced cardiac hypertrophy (TAC + placebo), and G-CSF treated mice with induced cardiac hypertrophy (TAC + G-CSF).

Cumulative survival of G-CSF as well as saline treated mice was recorded for 4 weeks after initiation of treatment. Mortality increased continuously throughout the recorded 4 weeks in both groups. However, G-CSF treated groups showed a significantly improved survival (61.8% *versus* 79.2%; Fig.6).

## Discussion

In the present study, we wanted to investigate whether G-CSF is able to attenuate cardiac hypertrophy and fibrosis in a mouse model of pressure-induced LV hypertrophy induced by TAC. Our main findings were the following: (*i*) G-CSF administration significantly increased homing of VLA-4^+^ and c-kit^+^ BMCs to the hypertrophic heart after TAC procedure. (*ii*) mRNA and Protein levels of VCAM-1- the ligand of VLA-4 and SCF- the ligand of c-kit were increased after G-CSF stimulation in the hypertrophic myocardium. (*iii*) G-CSF significantly reduced the area of fibrosis compared to untreated controls. (*iv*) Functionally, G-CSF significantly improved survival and preserved LV function after TAC procedure.

In the last years, several studies focused on the endogenous repair mechanisms after mobilization of BMCs by cytokines, like G-CSF 9,11,18, parathyroid hormone 10,13,19,20 or erythropoietin 21,22 in ischaemic diseases like MI. It could be clearly demonstrated that the interaction of SDF-1 with its ligand CXCR4 plays a major role in the migration of BMCs into ischaemic cardiac tissue, which was associated with attenuated remodelling and improved function 12,23. However, in non-ischaemic cardiomyopathies like DCM the interaction of SDF-1 with CXCR4 does not seem to be the important driver of migration, because SDF-1 expression was not up-regulated in cardiac tissue of humans and mice with DCM 16,24.

In our study, G-CSF treatment after pressure-induced LV hypertrophy significantly increased the amount of VLA-4^+^ and c-kit^+^ BMCs in the heart, whereas the number of CXCR4^+^ BMCs remained unchanged. In line with these results, we detected increased mRNA and protein expression of two corresponding homing factors VCAM-1 – the ligand of VLA-4 and SCF – the ligand of c-kit in the hypertrophic heart. Recently, Brunner *et al*. were able to demonstrate in a murine model of virus-induced DCM that G-CSF-induced VCAM-1/VLA-4 interaction is crucial for the recruitment of circulating BMCs to the myocardium 24. Notably, they could not detect increased numbers of c-kit^+^ or enhanced expression of SCF, suggesting a different regulation of cytokine profiles after DCM and pressure-induced LV hypertrophy. In contrast to our findings, Szardien *et al*. did not detect significant migration of BMCs 8 weeks after pressure unloading and G-CSF treatment in mice. They used enhanced green fluorescent protein (eGFP)-transgenic mice to track and distinguish the cells of BM origin from sedentary cells in the myocardium 17. However, two methodological differences might explain the different findings. First, Szardien *et al*. did a debanding 8 weeks after TAC procedure, which completely changes the cellular composition in the myocardium compared to a chronic pressure overload 25. Secondly, they did histological analyses 2 weeks after G-CSF pulsing to evaluate a numerical difference in eGFP^+^ cells between study groups. According to previous studies the maximum of mobilization and homing of BMCs occurs early after cytokine treatment 11. Therefore, they might have missed the peak of cell infiltration within the myocardium.

In accordance with the functional data from Szardien *et al*., we found a significant preservation of LV function after G-CSF treatment compared to controls. Histologically, there was an attenuated cardiac remodelling reflected by diminished amount of fibrosis. VCAM-1 has been shown to be involved in inflammatory processes. Recently, it has been demonstrated that VCAM-1/VLA-4 signalling exerts cardioprotective effects through enhanced angiogenesis 26 as well as facilitating survival of cardiomyocytes by activation of Akt, ERK and p38 MAPK 27. Likewise, G-CSF treatment was able to increase the expression of myocardial anti-apoptotic proteins and decreased TUNEL^+^ nuclei after MI 28. In this regard, we were interested whether G-CSF affects apoptosis after TAC-induced hypertrophy. Histological analysis of the hypertrophic LV revealed a decreased number of TUNEL^+^ cells reflecting a trend towards diminished apoptosis after G-CSF treatment. Our data also indicate an important role for c-kit^+^ cells, which are attracted by increased SCF protein levels in hypertrophic hearts after G-CSF injections. Fazel *et al*. were able to demonstrate that MI increased the number of c-kit^+^ cells in ischaemic hearts. They used genetic tagging in bone marrow chimeric mice to track these cells back to a bone marrow origin. Intriguingly, the recruited c-kit^+^cells established a pro-angiogenic milieu in the infarct border zone by increasing VEGF and by reversing the cardiac ratio of angiopoietin-1 to angiopoietin-2 29. In this regard, SCF significantly increased matrix metalloproteinases (MMP-2 and MMP-9) mRNA in mouse liver cells 30. Szardien *et al*. demonstrated that the G-CSF induced degradation of ECM in their model of pressure unloading was caused by an increased activity of both MMP-2 and MMP-9, both physiological enzymes with collagen-degrading properties 31.

**Figure 7:**
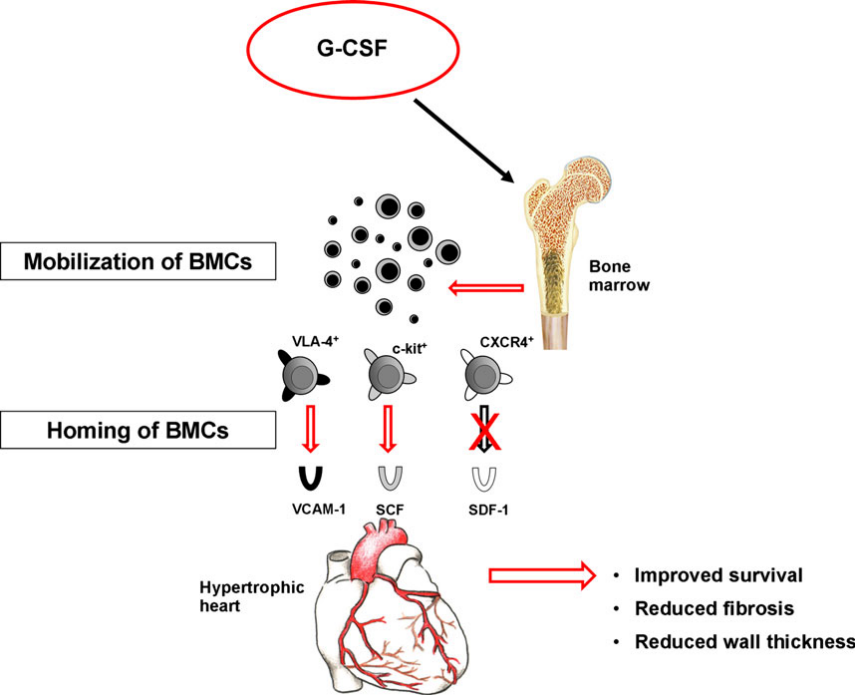
Suggested mechanism of G-CSF-mediated cardioprotection after TAC-induced hypertropy. G-CSF administration after TAC induces mobilization of stem cells from the BM to the peripheral blood. These stem cells migrate to the hypertrophic heart *via* the VLA-4/VCAM-1 and c-kit/SCF axes. The interaction of SDF-1 with CXCR4 seems not to be involved in this process.

In summary, our findings suggest a novel role of G-CSF in regressing fibrosis and preventing heart failure in pressure-induced cardiac hypertrophy, because of the modulation of cardiac cytokine profiles, which attract beneficial VLA-4^+^ and c-kit^+^ BMCs (Fig.7). However, it will be the goal of further studies to establish a direct link between cell migration and attenuation of cardiac remodelling after G-CSF treatment in hypertrophic hearts.
